# Imagining a brighter future: The effect of positive imagery training on mood, prospective mental imagery and emotional bias in older adults

**DOI:** 10.1016/j.psychres.2015.07.059

**Published:** 2015-11-30

**Authors:** Susannah E. Murphy, M. Clare O’Donoghue, Erin H.S. Drazich, Simon E. Blackwell, Anna Christina Nobre, Emily A. Holmes

**Affiliations:** a Oxford Centre for Human Brain Activity (OHBA), Department of Psychiatry, University of Oxford, Oxford, UK; b MRC Cognition and Brain Sciences Unit, Cambridge, UK; c Department of Clinical Neuroscience, Karolinska Institutet, Stockholm, Sweden

**Keywords:** Cognitive Bias Modification, Emotion bias, Optimism, Positive affect, Ageing, Mental imagery, Vividness, Cognitive training

## Abstract

Positive affect and optimism play an important role in healthy ageing and are associated with improved physical and cognitive health outcomes. This study investigated whether it is possible to boost positive affect and associated positive biases in this age group using cognitive training. The effect of computerised imagery-based cognitive bias modification on positive affect, vividness of positive prospective imagery and interpretation biases in older adults was measured. 77 older adults received 4 weeks (12 sessions) of imagery cognitive bias modification or a control condition. They were assessed at baseline, post-training and at a one-month follow-up. Both groups reported decreased negative affect and trait anxiety, and increased optimism across the three assessments. Imagery cognitive bias modification significantly increased the vividness of positive prospective imagery post-training, compared with the control training. Contrary to our hypothesis, there was no difference between the training groups in negative interpretation bias. This is a useful demonstration that it is possible to successfully engage older adults in computer-based cognitive training and to enhance the vividness of positive imagery about the future in this group. Future studies are needed to assess the longer-term consequences of such training and the impact on affect and wellbeing in more vulnerable groups.

## Highlights

• Older adults are able to engage successfully in computer-based Cognitive Bias Modification (CBM) training.
• Imagery CBM increased the vividness of imagery of future positive events in older adults.
• Both imagery CBM and control training improved mood and optimism in older adults.

## 1 Introduction

Older adulthood is associated with lower levels of negative affect (Charles and Carstensen, 2010) and lower rates of depression (Blazer, 2003). Consistent with this, there is a well documented age-related “positivity effect”, whereby older adults show an increased preference for positive over negative information in attention and memory (Reed et al., 2014). The importance of such positive affect for physical and cognitive health outcomes in older adults is increasingly apparent.

Higher levels of positive affect are associated with better concurrent and future health prospects, including reduced mortality (Chida and Steptoe, 2008; Lyyra et al., 2006) and reduced risk of coronary heart disease (Kubzansky and Thurston, 2007) and stroke (Ostir et al., 2001). These effects may be particularly relevant to older adulthood, a time when the accumulation of risk factors coupled with the ageing process contributes to a high incidence of chronic disease (Steptoe and Wardle, 2005). Importantly, the association between positive affect and health outcomes appears to be independent of factors such as smoking, body mass or socioeconomic status (Steptoe and Wardle, 2005). Indeed there is evidence of a direct effect of positive affective state on health-related biology, such as lower salivary cortisol, improved cardiovascular function (including blood pressure and heart rate) and immune system function (Dockray and Steptoe, 2010), which strengthens the argument that positive affect may be directly relevant to resilience to physical illness.

Positive affect has also been associated with improved cognitive function (Isen, 2008). There is evidence that positive mood improves performance across a range of cognitive tasks, including working memory (Yang et al., 2013) and rule described category learning (Nadler et al., 2010). In older populations, positive social networks have been shown to be protective against cognitive decline and dementia (Fratiglioni et al., 2000) and positive emotions experienced during these interactions are thought to be one reason for this (Charles and Carstensen, 2010).

Given this important role of positive affect in physical and cognitive health, it is interesting to consider if there are interventions that might be useful in further boosting positive affect in older adults. One possible approach comes from computer-based cognitive training paradigms, referred to as “cognitive bias modification”, that are designed to re-train dysfunctional biases in thinking (MacLeod et al., 2009; MacLeod and Mathews, 2012). Over the past decade there has been increasing interest in the therapeutic potential of cognitive bias modification in the prevention or treatment of mood disorders (Woud and Becker, 2014). In particular, a mental imagery-based cognitive bias modification approach has been shown to increase state positive affect in healthy young adult volunteers (Holmes et al., 2008a, 2009, 2006; Nelis et al., 2013) and dysphoric individuals (Pictet et al., 2011). As such, imagery cognitive bias modification may be a useful starting point in developing a cognitive training approach to boost positive affect in older adults.

In imagery cognitive bias modification, individuals are trained to automatically imagine positive resolutions of ambiguous information (Holmes et al., 2006, 2008c). Participants are required repeatedly to form positive images in response to ambiguous scenarios and pictures. This approach draws on evidence that mental imagery has a particularly powerful impact on enhancing positive emotion (Arntz et al., 2005; Holmes and Mathews, 2005, 2010; Holmes et al., 2006; Nelis et al., 2012). Recently, studies in clinically depressed individuals have demonstrated a decrease in negative interpretation bias on the Scrambled Sentences Task (a measure of biases in the interpretation of ambiguous information; Lang et al., 2012; Torkan et al., 2014), symptoms of depression (Lang et al., 2012; Torkan et al., 2014) and anhedonia (in the absence of a between-group effect on symptoms of depression; Blackwell et al., 2015), following imagery cognitive bias modification. Such evidence supports the use of imagery cognitive bias modification as a cognitive training approach that may hold promise as a low-intensity computer-based treatment tool in depression.

While boosting positive affect in older adults may be useful in itself, the practise in generating positive imagery inherent in imagery cognitive bias modification may also provide an additional route to benefits in this population. There appears to be a link between particular aspects of mental imagery and optimism, the tendency to have generalised positive expectancies about the future. Specifically, higher levels of optimism are associated with the ability to imagine positive future events more vividly (Blackwell et al., 2013). One possibility is that the ability to vividly imagine positive events in the future may lead to increased optimism. Simulation of possible future events, for example via mental imagery, is an important aspect of future-oriented thinking (e.g. Schacter et al., 2007), and the accessibility and clarity of such simulations may have an impact on how likely someone feels the events are to happen (Sharot et al., 2007; Szpunar and Schacter, 2013). Therefore, if, when someone simulates future events, they experience vivid mental images of positive possibilities, they may have more positive expectancies about the future, i.e. be more optimistic. Optimism is associated with a range of positive health outcomes in older adult populations, including reduced risk of future cardiovascular disease (Giltay et al., 2006) and even reduced rate of death (Giltay et al., 2004). If repeated practise in generating positive imagery during imagery cognitive bias modification could increase optimism in older adults via increased positive imagery vividness, this could confer benefits. However, it is currently not clear whether engaging in imagery cognitive bias modification can, in fact, lead to increased positive future imagery vividness, and whether such an increase in vividness would have a downstream effect on optimism.

The aim of this study was to investigate whether imagery cognitive bias modification is effective in boosting positive affect in a general population sample of older adults. To investigate this, older adults were randomised to receive 4 weeks of computerised imagery cognitive bias modification or a control training condition. They were assessed at baseline, at the end of training (week 4) and at a one-month follow-up (week 8). Consistent with previous studies in younger populations, it was hypothesised that imagery cognitive bias modification would increase positive affect (as measured by the Positive and Negative Affect Scale, PANAS; Watson et al., 1988). It was also hypothesised that imagery cognitive bias modification would increase the vividness of positive imagery about the future (measured using the Prospective Imagery Task, PIT; Stöber, 2000) and optimism (as measured by the Life Orientation Test-Revised, LOT-R; Scheier et al., 1994). Finally, it was hypothesised that imagery cognitive bias modification would decrease negative interpretation bias (measured using the Scrambled Sentences Test, SST; Wenzlaff and Bates, 1998), and improve scores on questionnaire measures of anxiety and quality of life.

## 2 Methods

### 2.1 Participants

A total of 81 healthy older adults (aged 60–80) were recruited from the community via local media and public advertisements. Four participants withdrew before completing the study due to bereavement, poor health, or difficulty travelling to the assessment centre, leaving a final sample of 77 participants. All participants were fluent in English, had normal or corrected-to-normal vision and hearing, and scored ≥26 on the Mini-Mental State Examination (Folstein et al., 1975). Participants had no current psychiatric disorder (with the exception of two participants who met criteria for specific phobia) or neurological diagnosis, and were taking no psychoactive medications. Participants were randomly assigned to either the “positive imagery” or “control” training condition (see Positive Imagery Training below). All participants provided written informed consent before taking part, and the study was approved by Oxford Central University Research Ethics Committee. Participants were paid £10 per hour for their time (up to £200) and travel expenses were reimbursed.

### 2.2 Procedure

Following a preliminary telephone screening, participants were invited for a face-to-face screening interview, in which they provided a brief medical history and completed the Structured Clinical Interview for DSM-IV (First et al., 1997), the Mini-Mental State Exam (MMSE; Folstein et al., 1975) and the Beck Depression Inventory – Second Edition (BDI–II; Beck et al., 1996). Eligible participants then completed a baseline assessment (see Section 2.3 below). Following the baseline assessment, participants were randomly allocated to one of two training conditions. Randomisation of participants to a training group was performed by the study Chief Investigator (SEM), who otherwise had no contact with the participants. Randomisation was stratified by scores on the BDI–II to ensure mood was balanced between training groups. Participants then began the training programme, which consisted of an introductory session, followed by 12 sessions over four weeks (see Positive Imagery Training below). Participants completed the training sessions in a quiet room in the Department of Psychiatry, University of Oxford in groups of between one and eight participants (typically a mixture of those in imagery and control training groups). Each participant sat at an individual desk with a laptop and headphones to complete the training. They were instructed not to talk to the other participants during the training sessions and not to discuss the content of the training with other participants for the duration of the study. On completion of the training, participants repeated the assessment (‘Post-training Assessment’) and had a Magnetic Resonance Imaging (MRI) and Magnetoencephalography (MEG) scan. Neuroimaging data are reported elsewhere. Four weeks after the final training session, participants completed the assessment for a third time (‘Follow-up Assessment’). Each participant was assigned a researcher who was unblind to the participant’s training condition. This researcher administered the initial training session and was available to the participant if they had any questions during the four weeks of training. A second researcher, who was blind to the participant’s training condition, conducted and scored the post-training and follow-up assessments.

### 2.3 Assessment

Participants completed the assessment at baseline, immediately post-training and at follow-up (4 weeks after the end of training). The assessment consisted of a battery of questionnaire measures and cognitive tasks designed to measure prospective imagery and emotional bias. Most participants completed the questionnaire measures at home (via a web-based survey program) before coming into the research centre to complete the cognitive tasks. If this was not possible (for example, if the participant did not have access to the internet at home), the questionnaires were completed online at the research centre at the start of the assessment.

#### 2.3.1 Questionnaire measures

Participants were asked to complete a range of self-report questionnaires measuring mood (BDI-II), state positive and negative affect (Positive and Negative Affect Scale, PANAS; Watson et al., 1988), trait anxiety (State-Trait Anxiety Inventory, STAI; Spielberger et al., 1983), optimism (Life Orientation Test Revised, LOT-R; Scheier et al., 1994), neuroticism (Eysenck Personality Questionnaire Neuroticism sub-scale, EPQ-N; Eysenck et al., 1985), quality of life (EuroQol-ED, EQ-5D; Kind, 1996) and tendency to use imagery (Spontaneous Use of Imagery Scale, SUIS; Reisberg et al., 2003).

#### 2.3.2 Prospective imagery test (PIT; based on Stöber, 2000)

The PIT is a measure of ability to generate mental images about future events. Participants are instructed to imagine 10 negative future scenarios (e.g. “you will have a serious disagreement with a good friend”) and 10 positive future scenarios (e.g. “people will admire you”), and rate the ‘vividness’ of each image on a 5-point Likert scale, from 1 (*no image at all*) to 5 (*very vivid*). Participants also rated their perceived “likelihood” of each event occurring in the near future, from 1 (*not at all likely to occur*) to 5 (*extremely likely to occur*), and the extent to which they felt that they were “experiencing” each event whilst imagining it, from 1 (*not at all*) to 5 (*completely*). Average scores are calculated for positive and negative items separately, for each of the three scales. The same twenty scenarios were used at all three behavioural assessments.

#### 2.3.3 Scrambled sentences task (SST, Wenzlaff and Bates, 1998)

The SST is a measure of negative interpretive bias, where participants use five of the six words in a scrambled sentence (e.g. ‘myself in disappointed am confident I’) to form either a positive sentence (e.g. ‘I am confident in myself’) or a negative sentence (e.g. ‘I am disappointed in myself’). Participants are given four minutes to unscramble a list of 20 sentences, under cognitive load (remembering a 6-digit number). A negativity score is calculated as the proportion of correct negative items. Two equivalent sentences lists, each with 20 novel sentences, and two equivalent 6-digit cognitive load numbers were used. All participants completed version A at baseline and follow-up assessments, and version B at post-training.

### 2.4 Positive imagery training

The imagery intervention was based on that previously reported (e.g. Lang et al., 2012), but with an extended schedule of 12 sessions over a 4-week period (following Blackwell et al., 2015) in order to have a higher “dose” of training and longer time-frame available for changes in the outcomes of interest. Six sessions were auditory, in which participants listened to short descriptions of everyday situations and were instructed to imagine themselves in the described scenario ‘as if actively involved and seeing it through their own eyes’. The outcome of each scenario was initially ambiguous, but all descriptions resolved positively. Six sessions were in a picture-word format, where participants were shown ambiguous photos of everyday scenes paired with a few words that resolved the scene in a positive way. Participants were instructed to generate a mental image incorporating the picture and words. In both auditory and picture-word sessions, participants were asked to rate ‘how vividly could you imagine the described scenario?’, from 1 (*not at all vivid*) to 5 (*extremely vivid*) after each trial. Each of the 12 sessions started with reminder instructions and a practise example, followed by 8 blocks of 8 trials (64 trials per session) with self-paced breaks between blocks. Each session took approximately 20 min. Stimuli were not repeated, therefore each participant was presented with 416 unique auditory stimuli and 416 unique picture-word stimuli in total across the initial practise session and 12 training sessions. The stimuli were adapted from those used by Blackwell and colleagues (2015) to make them suitable for an older age group. This adaptation was carried out in consultation with a focus group of older adults, who were asked to give feedback on the relevance of the scenarios to their everyday lives and other factors, such as ease of use of the program, clarity of the stimuli, and how understandable the task was. The discussion in the focus group indicated that the computer task was easy to use and that the majority of the original scenarios were relevant to the older age group. However, there were some ‘themes’, such as work concerns, that were identified as being less relevant for this age group. The focus group were then asked to give examples of their daily activities and these were used to generate new/adapted scenarios. Following this feedback, 120 of the training scenarios were modified.

The control condition was identical, except the active ‘imagery’ component was removed, as was the contingency between ambiguity and positivity. In auditory sessions, participants were instructed to focus on the words and meanings of the descriptions, and rate ‘how difficult was it to understand the meaning of the description?’ from 1 (*not at all difficult)* to 5 (*extremely difficult).* In picture-word sessions, participants were asked to generate a sentence combining the picture and words, and asked to rate ‘how difficult was it to generate a sentence?’ from 1 (*not at all difficult)* to 5 (*extremely difficult).* Half of the auditory scenarios resolved positively, whilst half had negative resolutions. Similarly, half of the pictures were paired with positive captions, whilst half had negative captions.

All participants completed an introductory session before starting the 12 training sessions. The introductory sessions began with a brief standardised introduction to the intervention (~15 min), administered by the researcher. Imagery participants were given an introduction to mental imagery and practiced generating mental images. Control participants were given an introduction to verbal processing, and practise with verbal processing. Participants then completed a practise session of their assigned training condition, with guidance from the researcher. This practise session consisted of 4 sets of 8 auditory trials, and 4 sets of 8 picture-word trials.

At post-training, participants completed ratings of their engagement with the intervention over the four weeks, to measure adherence with the experimental manipulation (imagery or verbal). These included ratings of use of imagery: ‘How much were you imagining the situations from through your own eyes, as if actively involved (i.e. from a personal point of view) as you were listening to the scenarios?’ and ‘How much were you imagining the picture-word combinations through your own eyes, as if actively involved (i.e. from a personal point of view)?’, and use of verbal processing: ‘How much did you find yourself verbally analysing the meaning of the scenarios as you were listening to them?’ and ‘How much did you find yourself thinking verbally about the picture-word combinations?’, on a scale from 1 (*not at all*) to 9 (*all the time*).

### 2.5 Data analysis

Baseline measures were compared between groups using independent *t*-tests for continuous data and chi-square tests for categorical data. Where Levene’s test indicated unequal variances, Welch's t-test was used. Change in questionnaire measures over time was assessed using a (2×3) split-plot analysis of variance (ANOVA) with the between-subject factor of training group and the within-subject factor of time of assessment (i.e., baseline, post-training and follow-up).

Data from the cognitive tasks were analysed using split-plot ANOVAs with training group as the between-subject factor and time of assessment and within-subject factors of task conditions. Where the assumption of sphericity was violated, degrees of freedom were corrected using Greenhouse-Geisser estimates of sphericity. Significant interactions were explored further using independent samples t-tests.

## 3 Results

### 3.1 Behavioural measures

#### 3.1.1 Baseline measures

The groups were well matched in terms of age, education, gender, ethnicity, marital status and employment status. There were also no significant differences between the two groups in terms of MMSE score and self-reported use of imagery at baseline (see Table 1). Table 2 presents the correlations between baseline measures.

#### 3.1.2 Compliance with training

Compliance with the training schedule was extremely good, with 96% of the participants completing all 12 training sessions. There was no difference between the two groups in the number of sessions completed (imagery condition: mean=12, SD=0; control condition: mean=11.9, SD=0.7; *t*(40)=1.15, *p*=0.26).

On completion of the training program, participants in the imagery condition reported using imagery significantly more in the intervention sessions than participants in the control condition (imagery condition: mean=7.8, SD=1.1; control condition: mean=5.0, SD=2.2; *t*(56.9)=7.26, *p*<0.001). Participants in the imagery condition reported using verbal analysis significantly less in the intervention sessions than participants in the control condition (imagery condition: mean=3.6, SD=2.0; control condition: mean=7.1, SD=1.3; *t*(58.8)=8.82, *p*<0.001). One participant did not complete the follow-up behavioural assessment, due to poor health so data reported at this time point is for 76 participants.

#### 3.1.3 Questionnaire measures

Repeated-measures ANOVAs were conducted to assess the effect of training on questionnaire measures. There were no significant main effects of group or interactions between time×group across all measures. However, there were significant main effects of time on the following measures: *BDI–II* [F(2, 150)=7.51, *p*=0.001, η^2^=0.09], *PANAS* negative subscale [F(1.8, 117.7)=15.22, *p*<0.001, η^2^=0.19], *TAI* [F(1.8, 135.7)=17.10, *p*<0.001, η^2^=0.19], *LOT-R* [F(2, 150)=4.56, *p*=0.01, η^2^=0.06], and *EPQN* [F(1.8, 136.8)=3.83, *p*=0.03, η^2^=0.05]. These significant effects of time reflected decreased scores on the BDI-II, PANAS negative subscale, TAI and EPQN and increased scores on the LOT-R, which is consistent with both groups reporting improved mood post-training (see Table 3). There were no significant effects of time or time×group interactions for the PANAS positive subscale (Time: F(2, 132)=2.87, *p*=0.06, η^2^=0.042; Time×Group: F(2, 132)=0.95, *p*=0.39, η^2^=0.014) or EQ-5D (Time: F(1.8, 136.9)=1.19, *p*=0.31, η^2^=0.016; Time×Group: F(1.8, 136.9)=0.95, *p*=0.38, η^2^=0.013).

#### 3.1.4 Prospective imagery task

One participant did not complete the PIT at baseline. Therefore the data from 75 participants are reported here (36 imagery group; 39 control group).

**Fig. 1:**
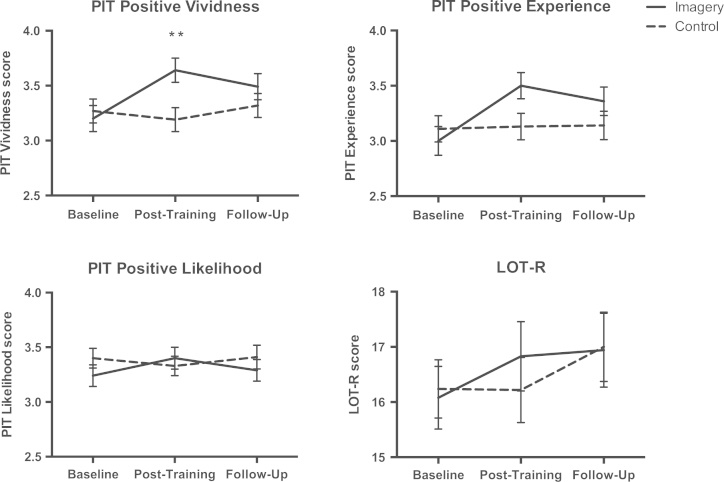
Prospective Imagery Test and Optimism scores on the three sub-scales of the Prospective Imagery Test (PIT) and on the Life Orientation Test – Revised (LOT-R) at baseline, post-training and follow-up assessments. Mean scores for the imagery (solid line) and control (dashed line) training groups are displayed. Error bars represent SEM. ** *p*<0.01.

Vividness scores: There was a significant interaction between time×valence×group for the ratings of vividness [F(2, 146)=4.22, *p*=0.02, η^2^=0.06]. In order to explore this, ratings for positive and negative scenarios were analysed in separate 2-way ANOVAs, with time and group as factors. For the positive scenarios (Fig. 1), there was a significant interaction between time and group [F(1. 8, 127.9)=7.27, *p*=0.002, η^2^=0.09]. Independent samples *t*-tests revealed that this interaction was driven by significantly increased vividness ratings in the imagery group at the post-training time point compared with the control group [*t*(73)=−1.13, *p*=0.006, d=0.65]. There was no significant difference between groups at baseline [*t*(73)=−0.48, *p*=0.64, *d*=−0.11] or follow-up [t(73)=1.04, *p*=0.30, *d*=0.24]. For the negative scenarios, there was no significant interaction between time and group [F(2, 146)=0.95, *p*=0.39, η^2^=0.013].

Likelihood scores: There was a marginally significant interaction between time×valence×group for the ratings of likelihood [F(2, 146)=2.71, *p*=0.07, η^2^=0.04]. In order to explore this, ratings for positive and negative scenarios were analysed in separate 2-way ANOVAs, with time and group as factors. For the positive scenarios (Fig. 1), there was a significant interaction between time and group [F(2, 146)=3.12, *p*=0.047, η^2^=0.04]. However, independent samples t-tests showed that the two groups were not significantly different in their likelihood ratings of the positive scenarios at any of the three time points (Baseline: *t*(73)=−1.13, *p*=0.26, *d*=−0.26; Post-training: *t*(73)= 0.56, *p*=0.58, *d*=0.13; Follow-up: *t*(73)=−0.79, *p*=0.43, *d*=-0.18). Again, there was no significant interaction between time and group for the negative scenarios [Time*group: F(2, 146)=0.12, *p*=0.89, η^2^=0.002].

Experiencing scores: There was no significant interaction between time×valence×group for the experiencing ratings [F(2, 146)=0.65, *p*=0.52, η^2^=0.009]. However, there was a significant time×group interaction [F(2, 146)=5.074, *p*=0.007, η^2^=0.65]. Separate analysis of the two training groups revealed that, in the imagery group, there was a significant effect of time [F(2,70)=7.921, *p*=0.001, η^2^=0.185], with increased ratings of experiencing at both post-training and follow-up compared with baseline. In the control group, there was no significant effect of time [F(2,76)= 0.01, *p*=0.99, η^2^=0.000].

#### 3.1.5 Scrambled sentences task

For the Scrambled Sentences Task, there was no main effect of time [F(1.8,129.7)=0.54, *p*=0.6, η^2^=0.008] or group [F(1,71)=1.44, *p*=0.2, η^2^=0.02], and no significant interaction between time and condition [F(1.8,129.7)=0.09, *p*=0.9, η^2^=0.001] (see Table 3).

## 4 Discussion

The aim of the current study was to investigate whether a simple computer-training program could increase positive affect in a sample of older adults. More specifically, we investigated the effect of imagery cognitive bias modification on mood, prospective imagery, optimism and emotional bias in an unselected sample of older adults. Overall, mood improved over time, with both groups reporting a decrease in negative affect and trait anxiety. Participants also showed an increase in optimism over time regardless of training condition. However, there was no difference between the two groups in reported change in mood, and no improvement in positive affect in either group. Imagery cognitive bias modification did significantly increase the vividness of positive imagery about the future compared with the control training. Contrary to our hypothesis, there was no difference between the training groups in interpretation bias, as measured by the Scrambled Sentences Task.

Unexpectedly, we found that both training groups showed a small improvement in mood, with a self-reported decrease in negative mood, trait anxiety, depression and neuroticism, and an increase in optimism. This finding is in contrast to a number of studies in younger age adults in which imagery cognitive bias modification was found to have a specific effect on mood compared with a control condition, with an increase in positive affect (Holmes et al., 2008a, 2009) and a reduction in depression symptoms (Lang et al., 2012; Torkan et al., 2014). However, it is worth noting that these previous studies investigated the effects of imagery cognitive bias modification over a shorter timescale (e.g. positive affect immediately after a single session of training, or symptoms of depression following one week of training). A recent study, using a four-week training schedule and the same control condition as the current study, did not find a predicted between-group difference on reduction in symptoms of depression in a sample of 150 adults with current major depression (Blackwell et al., 2015). The authors suggest that in extending training schedules from brief to longer timescales, a number of factors that would not be apparent in single-session experimental studies may come into play. Thus a broader consideration of the mechanisms by which engaging in such extended cognitive training programmes could impact on outcomes will be a useful way forward.

Whilst the effects on mood that were seen in both groups in the current study were relatively small, they are interesting given that some other cognitive bias modification studies have also found that both active and control training groups show an improvement in mood (Blackwell et al., 2015; Carlbring et al., 2012; Salemink et al., 2014). In response to this, Salemink and colleagues (2014) suggested that a control condition with mixed valence stimuli (half positive and half negative), such as the one used in the present study, may still actively encourage an increase in positive interpretations. In such situations, there would be an expected increase in positive mood in both groups, which may mask any specific effect of the active training condition. However, in an experimental test of this idea, in which the 50/50 control condition was replaced with a 100% neutral control condition, there was still a general improvement in mood in both the active and control condition (Salemink et al., 2014). Therefore, it seems likely that the general improvement in mood seen in the current study may have been driven by non-specific factors, such as the enjoyment of taking part in a research study, increased social interaction with researchers and other study participants, cognitive stimulation, or expectation of training effects. The relatively small shifts in mood that were seen (i.e. approximately one point on the Beck Depression Inventory) are consistent with such an explanation. It may be that in our older adult sample these non-specific factors had a stronger effect than in previous studies in younger populations. In particular, the delivery of the training to participants in groups may have increased the social interaction associated with taking part in the study. This may have had a particularly strong non-specific effect on mood in our older adult sample, who may experience more social isolation than the younger samples included in previous studies. Therefore the potentially large impact of coming into the research centre 12 times over four weeks may mask any relatively subtle between-groups differences related to the actual content of the 20 minute training session. This delivery format may have also attracted a sample of older adults who would find the social interaction aspects of the study particularly appealing. Delivering the intervention to participants at home (as was the case in the studies by Lang et al. (2012) and Blackwell et al. (2015)), rather than the laboratory setting used in the current study, may be one way to reduce such non-specific effects in future studies. However, despite these potential limitations of delivering the training in the research centre, this approach did mean that the researchers were able to provide support with any questions or technical issues relating to the training, which may have contributed to the extremely high level of training compliance in this study. We were also able to assess whether older adults were able to engage with the computerised training, to determine whether web-based training, completed at home, would be feasible in this age group.

Despite the failure to see a specific effect of the imagery cognitive bias modification training on positive affect in the current study, there was a significant increase in the vividness of ratings of positive future events, and the extent to which participants experienced these images as if they were really happening, in the imagery-trained group compared with the control group. Such a finding is interesting in light of the evidence that the vividness of prospective positive imagery is associated with mood and optimism. For example, the ability to generate vivid mental images of positive future events has been shown to be significantly associated with higher scores on a self-report measure of optimism (Blackwell et al., 2013). Further, a number of studies have reported decreased vividness of imagery related to prospective positive events in people with higher levels of dysphoria (Holmes et al., 2008b) and depression (Morina et al., 2011; Stöber, 2000). However, we did not find that the increased vividness of positive future imagery in the imagery condition led to a relatively greater increase in optimism relative to the control condition. It may be that the increased ratings for imagery vividness on the prospective imagery task in the imagery condition simply reflect an inflation of these ratings due to, for example, demand effects following four weeks of making vividness ratings, rather than reflecting an actual increase in the vividness of imagery generated. However, it could also be that, if there is a causal link between vividness of positive future imagery and optimism, a sustained increase in vividness over a longer period of time is required to have a significant impact on optimism, which is seen as a very stable personality trait (Carver et al., 2010). Further, within the broader context of cognitive training interventions, it is often the case that people improve at the task they practise, but this does not transfer to other tasks or domains (Owen et al., 2010; Zelinski, 2009). It may be that this is what occurred in the case of participants in our imagery condition, and how to enhance such transfer effects is an important question for the field of cognitive training as a whole.

However, the effect of the imagery cognitive bias modification on positive future imagery is an interesting area to explore further. Importantly, the vividness of prospective imagery has also been shown to be predictive of future behaviour. A long history of research has demonstrated that mentally imagining future positive events can increase motivation, effort and behaviour directed at achieving them (Cantor et al., 1986; Karniol and Ross, 1996; Taylor et al., 1998). Although the effect of the intervention on behaviour was not directly measured in the current study, the increased vividness ratings of positive prospective imagery seen as a result of the training are nonetheless consistent with the idea that the imagery training had an impact on positive thinking and that this may have important implications for engagement in positive behaviours in older adults. Our understanding of the potential mechanisms involved could benefit from linking with findings emerging from the broader scientific literature of the beneficial effects of activation of neural representations of positive events (Dranovsky and Leonardo, 2015; Ramirez et al., 2015), and this will be useful to explore in future work.

Interestingly, the pattern of change in the vividness ratings was not mirrored in the pattern of change for the likelihood and experiencing ratings for the prospective imagery test: for likelihood ratings, there was no difference between the two training conditions at any time-point, whereas experiencing ratings increased more in the imagery compared to the control condition regardless of image valence. We would expect ratings of likelihood and experiencing to be closely related to vividness, and thus this differential effect of the training suggests that the relationships between these aspects of future-oriented imagery need further investigation. It is important to note that participants imagined the same scenarios at each assessment time point on the prospective imagery test, and in future it may be useful to test changes in imagery ability using novel scenarios counterbalanced across presentations.

These findings should be interpreted within the context of the older adult sample used. As well as being older than participants included in previous imagery cognitive bias modification studies, the sample in the present study had very low levels of depression and anxiety at baseline. Consistent with this, baseline negativity scores on the Scrambled Sentences Task were very low, and this may account for the failure to find an effect of imagery cognitive bias modification on this task, as there was little scope to further induce a positive bias. Given the ‘positivity effect’ that is well described in older populations, whereby older adults show an increased tendency to focus on positive compared to negative information (Reed et al., 2014; Steinman et al., 2013), it may be that a sample selected for lower mood may be necessary to see clearer effects of imagery cognitive bias modification on positive affect in this age group.

Nonetheless, the current study is a useful demonstration that it is possible to engage older adults in computer based cognitive training successfully, as shown by the high compliance in session attendance and completion, and to modify the vividness of positive imagery about the future in this group. It will be important to assess the longer term consequences of such training in future studies, for example on behaviour, and whether it has an impact on affect and wellbeing in more vulnerable groups.

## Figures and Tables

**Table 1:** Demographic details for participants aged 60–80 that completed the cognitive training

	Imagery (*n*=36)	Control (*n*=41)	Statistics
	M (SD)	M (SD)	
Age (years)	68.2 (6.9)	66.1 (4.9)	*p*=0.14
Years of education	15.8 (4.0)	17.0 (3.9)	*p*=0.18
MMSE	29.2 (0.9)	29.1 (0.8)	*p*=0.71
SUIS	38.1 (6.1)	35.9 (7.9)	*p*=0.19
	N (%)	N (%)	
Gender	23 (63.9%)	21 (51.2%)	*p*=0.26
Ethnicity white	35 (97.2%)	40 (97.6%)	*p*=0.37
Marital status			
Married	18 (50.0%)	24 (58.5%)	*p*=0.56
Cohabiting	2 (5.6%)	3 (7.3%)
Single	16 (44.4%)	13 (31.7%)
*Missing*	0 (0.0%)	1 (2.4%)
Employment status			
Current full time	5 (13.9%)	7 (17.1%)	*p*=0.93
Current part time	4 (11.1%)	6 (14.6%)
Retired	26 (72.2%)	27 (65.9%)
Unemployed	1 (2.8%)	1 (2.4%)
Highest qualification			
None	2 (5.6%)	2 (4.9%)	*p*=0.35
O-level	4 (11.1%)	3 (7.3%)
A-level	3 (8.3%)	4 (9.8%)
College/Professional	8 (22.2%)	10 (24.4%)
Undergraduate	11 (30.6%)	7 (17.1%)
Post-graduate	7 (19.4%)	15 (36.6%)
*Missing*	1 (2.8%)	0 (0.0%)

Abbreviations: *MMSE* – Mini-Mental State Exam; *SUIS* – Spontaneous Use of Imagery Scale. Independent sample t-tests conducted for age, years of education, MMSE and SUIS. Chi-square tests conducted for gender, ethnicity, marital status, employment status and highest qualification.

**Table 2:** Correlations between participant characteristics and outcome measures at baseline

Measure	1	2	3	4	5	6	7	8	9	10	11	12	13	14	15	16
1. Age	-	0.301**	-0.364**	-0.033	0.154	-0.176	0.033	-0.207^†^	0.202^†^	-0.109	-0.246^*^	-0.136	-0.047	-0.099	-0.094	0.110
2. BDI		−	-0.588**	0.535**	0.594**	-0.454**	0.451**	-0.444**	0.203†	-0.084	-0.357**	-0.105	0.090	0.171	0.090	0.340**
3. PANAS-P			−	-0.217†	-0.629**	0.425**	−0.278*	0.354**	-0.222^†^	0.252*	0.481**	0.333**	-0.026	-0.146	-0.001	-0.142
4. PANAS-N				−	0.595**	-0.326**	0.478**	-0.389**	0.234†	0.005	-0.168	-0.017	0.252*	0.432**	0.293*	0.259*
5. TAI					−	-0.717**	0.684**	-0.303**	0.070	-0.270*	-0.425**	-0.200†	0.123	0.407**	0.270*	0.427**
6. LOT-R						−	-0.630**	0.214†	-0.043	0.314**	0.467**	0.275*	-0.057	-0.151	-0.182	-0.326**
7. EPQ-N							−	-0.138	-0.108	-0.214†	-0.361**	-0.195†	0.058	0.221†	0.125	0.202†
8. EQ-5D								−	–0.066	-0.002	0.106	-0.039	-0.212†	-0.237*	-0.238*	-0.322**
9. SUIS									–	0.311**	-0.004	0.265*	0.313**	-0.122	0.231*	0.039
10. PIT-P-V										–	0.421**	0.817**	0.710**	0.090	0.498**	-0.082
11. PIT-P-L											–	0.472**	0.096	0.043	0.111	-0.109
12. PIT-P-E												–	0.607**	0.113	0.681**	0.011
13. PIT-N-V													–	0.419**	0.793**	0.206†
14. PIT-N-L														–	0.493**	0.295*
15. PIT-N-E															−	0.254*
16. SST																−

*Note*: BDI – Beck's Depression Inventory; PANAS – Positive and Negative Affect Scale, -P – Positive, -N – Negative; TAI – Trait Anxiety Inventory; LOT-R – Life Orientation Test Revisited; EPQ-N – Eysenck Personality Questionnaire Neuroticism Scale; EQ-5D – EuroQol (global health rating); SUIS – Spontaneous Use of Imagery Scale; PIT – Prospective Imagery Test, -P – Positive, -N – Negative, -V – Vividness, -L – Likelihood, -E – Experiencing, SST – Scrambled Sentences Task.

N=77 (BDI, TAI, LOT-R, EPQ-N, EQ-5D), *N*=76 (PIT), *N*=74 (SST), *N*=68 (PANAS)

† *p*<0.10

* *p*<0.05

** *p*<0.01

**Table 3:** Self-report mood measures before and after training

	**Baseline**	**Post-Training**	**Follow-Up**
*Mean (SD)*	*Mean (SD)*	*Mean (SD)*
BDI*			
*Imagery*	4.58 (3.37)	3.50 (2.99)	3.22 (2.47)
*Control*	4.93 (4.46)	3.83 (4.71)	3.71 (3.89)
**PANAS**			
*Positive Affect*			
*Imagery*	34.25 (6.12)	35.09 (5.22)	36.34 (5.61)
*Control*	33.94 (6.57)	34.69 (7.31)	34.61 (7.40)
Negative Affect*			
*Imagery*	13.66 (3.00)	12.66 (3.70)	12.03 (2.74)
*Control*	14.36 (4.10)	13.44 (3.78)	12.78 (3.38)
TAI*			
*Imagery*	32.25 (7.80)	29.89 (8.62)	28.42 (8.57)
*Control*	33.88 (8.96)	32.88 (9.09)	31.85 (9.30)
LOT-R*			
*Imagery*	16.08 (3.20)	16.83 (3.98)	16.94 (3.66)
*Control*	16.24 (3.56)	16.22 (3.62)	17.00 (4.36)
EPQ-N*			
*Imagery*	2.49 (2.48)	2.26 (2.55)	1.86 (2.52)
*Control*	2.88 (3.15)	2.85 (3.21)	2.59 (3.41)
**EQ-5D**			
*Imagery*	86.39 (10.20)	85.06 (10.38)	87.89 (9.00)
*Control*	83.68 (12.30)	81.88 (14.04)	82.17 (14.11)
**SST**			
*Imagery*	0.13 (0.15)	0.13 (0.11)	0.12 (0.17)
*Control*	0.17 (0.17)	0.18 (0.15)	0.15 (0.18)

Abbreviations: BDI – Beck's Depression Inventory; PANAS – Positive and Negative Affect Scale; TAI – Trait Anxiety Inventory; LOT-R – Life Orientation Test Revisited; EPQ-N – Eysenck Personality Questionnaire Neuroticism Scale; EQ-5D – EuroQol-ED (global health rating); SST – Scrambled Sentences Task.

* denotes significant main effect of ‘time’ in repeated measures ANOVA.
